# Geographical Variations in Virulence Factors and Antimicrobial Resistance Amongst Staphylococci Isolated From Dogs From the United Kingdom and Romania

**DOI:** 10.3389/fvets.2020.00414

**Published:** 2020-07-21

**Authors:** Ozana M. Hritcu, Vanessa M. Schmidt, Shebl E. Salem, Iuliana E. Maciuca, Ramona F. Moraru, Irina Lipovan, Mihai Mareş, Gheorghe Solcan, Dorina Timofte

**Affiliations:** ^1^Faculty of Veterinary Medicine, University of Agronomical Sciences and Veterinary Medicine, Iaşi, Romania; ^2^Department of Small Animal Clinical Science, Institute of Infection, Veterinary and Ecological Sciences, University of Liverpool, Wirral, United Kingdom; ^3^Department of Infection Biology, Institute of Infection and Global Health, University of Liverpool, Wirral, United Kingdom; ^4^Department of Surgery, Faculty of Veterinary Medicine, Zagazig University, Zagazig, Egypt; ^5^Department of Veterinary Anatomy Physiology and Pathology, Institute of Infection, Veterinary and Ecological Sciences, University of Liverpool, Liverpool, United Kingdom

**Keywords:** dog, pyoderma, *Staphylococcus pseudintermedius*, antimicrobial resistance, Romania, UK

## Abstract

The objective of this study was to compare virulence and resistance factors of mucosal and cutaneous staphylococci from dogs with pyoderma in the UK and Romania, two countries with different approaches to antimicrobial use in companion animals. Staphylococcal isolates (*n* = 166) identified to the species level as being *Staphylococcus pseudintermedius* or coagulase negative (CoNS) were analyzed for their antimicrobial resistance (AMR) profile and presence of resistance and virulence genes. Of the investigated isolates, 26 were methicillin-resistant *S. pseudintermedius* (MRSP), 89 were methicillin-susceptible *S. pseudintermedius* (MSSP) and 51 were coagulase negative staphylococci (CoNS). A significantly larger number of isolates originating from Romania were resistant to clindamycin, tetracycline, and chloramphenicol compared to the UK isolates (*P* < 0.05). Resistance to amoxicillin-clavulanic acid, gentamicin, and trimethoprim-sulphamethoxazole was more evident in UK isolates. Fusidic acid resistance was common in *Staphylococcus* spp. isolates from both countries. Most isolates carried virulence factors associated with *siet* (exfoliative toxin) and *luk* (leucocidin) genes. All MRSP UK isolates exhibited fusidic acid resistance genes whilst this was very rare in the MRSP isolates from Romania. The chlorhexidine resistance gene *qacA/B* was frequently identified in CoNS isolates from the UK (*P* < 0.001). The current study documented differences in antimicrobial resistance profiles of *Staphylococcus* spp. isolates from dogs in two geographical locations in Europe, which could reflect differences in antimicrobial prescribing patterns. The study also highlights the need for further studies and interventions on antimicrobial use, prescribing patterns and AMR surveillance in companion animals in Romania.

## Introduction

*Staphylococcus pseudintermedius*, formerly *Staphylcoccus intermedius* (1), is considered the main coagulase positive staphylococci (CoPS) commensal of canine skin and mucosa (2). It is often present with other mucosal and skin commensals such as coagulase negative staphylococci (CoNS) and other CoPS such as *Staphylococcus aureus* and *Staphylococcus schleiferi* (3). *S. pseudintermedius* may also be pathogenic and is considered the main cause of bacterial pyoderma in dogs (4). Systemic or topical antibiotics or antiseptics may be employed to treat dogs with pyoderma (4) depending on the underlying primary diagnosis.

The pathogenesis and clinical importance of *S. pseudintermedius* is determined by the virulence and antimicrobial resistance factors that it possesses or may acquire from other bacteria in co-colonization. *S. pseudintermedius* possesses a number of enzymes (coagulase, thermonuclease, proteases), toxins (cytotoxin, exfoliative toxin, enterotoxin, leukocidins, haemolysin) and adhesion factors (clumping factor, protein A, biofilm forming proteins) which can facilitate its pathogenicity (5). Furthermore, acquisition of antimicrobial resistance determinants makes infections with such bacteria more difficult to treat. Carriage of the *mec*A gene on the staphylococcal cassette chromosome *mec* (SCC*mec*) confers resistance to all β-lactam antimicrobials (methicillin-resistant *S. pseudintermedius*) (2) and isolates are often multidrug resistant (MDR, exhibiting resistance to at least 1 agent in ≥3 antimicrobial categories) (6). In addition, CoNS are commonly antimicrobial resistant, including methicillin resistance (7), and thought to be the original source of *mec*A gene in *S. aureus* (8). Resistance to topical antimicrobials and antiseptics has been frequently reported amongst human *S. aureus* and CoNS isolates (9), but only rarely reported amongst canine *S. pseudintermedius* isolates (10–12).

Romania has a high prevalence of antimicrobial resistance (AMR) and there is limited information on AMR surveillance in bacterial populations from humans and animals. A recent study which analyzed global antibiotic consumption patterns over time, showed that Romania ranked 6th in the world for the consumption of daily doses of antibiotics per inhabitant (13). Even more concerning, another study has shown that Romania has the highest rate of non-prescription use of antibiotics in human populations in Europe with 20% of antibiotics being released without prescription (14). In addition, European data collected through the European Antimicrobial Resistance Surveillance Network (EARS-Net) shows that Romania had the highest occurrence of methicillin-resistant *S. aureus* (MRSA) invasive bloodstream infections in 2017 (44.4%) compared to countries such as UK (7.2%) or Norway, Sweden and the Netherlands where it can be as low as 1% (15). Although there is no similar data available on antimicrobial resistance surveillance or antimicrobial use in companion animals in Romania, we can hypothesize that a similar trend with high antibiotic consumption mirrored by high AMR prevalence is present in the companion animal population. We therefore aimed to investigate and compare mucosal and cutaneous methicillin-susceptible *S. pseudintermedius* (MSSP), methicillin-resistant *S. pseudintermedius* (MRSP), and CoNS isolated from dogs with pyoderma, from two different European geographic areas with likely very different approaches to antimicrobial use in companion animals (Eastern Romania and the United Kingdom). For this, the isolates were analyzed for antimicrobial resistance phenotypes, genotypic antimicrobial and antiseptic resistance markers, and for the carriage of virulence factors.

## Materials and Methods

### Sample Collection and Isolates Identification

The study population included dogs admitted to two referral veterinary hospitals (one based in Romania and the other in the UK). Dogs were examined in the Dermatology Department of each hospital and were recruited onto the study if they were diagnosed with secondary staphylococcal pyoderma based on clinical signs and cytological evidence (4). The study group was heterogeneous, containing purebred and crossbreed dogs, males and females, with no predominance. The patients' age varied from 6 months to 8 years and the sampling period was June 2014–January 2016. Previous treatments with antimicrobials could not be determined with accuracy for the patients in Romania due to the lack of data provided by the owners or previous veterinarians. Ethical approval for the study was obtained from the University of Liverpool ethics committee in June 2011, and the Romanian Faculty of Veterinary Science Ethics and Deontology Committee in September 2014.

*Staphylococcus* spp. isolates were obtained by sampling the anterior nares and/or perineal skin (3) using sterile swabs with Amies transport media (FLmedical, Italy). The swabs were inoculated onto Columbia Agar Base (CAB; Oxoid, Basingstoke, UK) and Mannitol Salt Agar (MSA; Oxoid, Basingstoke, UK) and incubated aerobically at 35°C for 24 h. Isolates with colony morphology typical of staphylococci were selected from all plates, sub-cultured onto CAB and incubated aerobically overnight at 35°C. All fresh cultures from CAB were subjected to Gram-staining and catalase test. Rabbit plasma agglutination test was performed on all isolates to detect free coagulase where *S. aureus* ATCC®25923 and *S. epidermidis* ATCC®12228 were used as positive and negative controls, respectively. Species identification was confirmed by PCR amplification of the *nuc* gene for *S. pseudintermedius* (16) and by MALDI-TOF MS (Bruker, Bremen, Germany) for the CoNS-UK isolates but was not available for identification of CoNS-RO isolates.

### Antimicrobial Susceptibility Testing

Disc diffusion testing was performed on all staphylococcal isolates. Two Mueller Hinton agar plates were inoculated with each isolate homogenized in sterile distilled water (0.5 McFarland standards) for semi-confluent growth using a cotton swab and a rotary plating device. Twelve antimicrobial disks were then applied to the surface including: oxacillin (1 μg), cefalexin (30 μg), cefovecin (30 μg), amoxicillin-clavulanic acid (30 μg), clindamycin (2 μg), trimethoprim-sulfamethoxazole (1.25/23.75 μg), gentamicin (10 μg), tetracycline (30 μg), chloramphenicol (30 μg), enrofloxacin (5 μg), and fusidic acid (10 μg) (Oxoid, Basingstoke, UK and the media from LabM Ltd, Bury, UK). The plates were incubated aerobically at 35°C for 18–24 h. Interpretation of the tests was based on the Clinical and Laboratory Standards Institute guidelines for veterinary pathogens or human-derived interpretive standards when veterinary interpretative criteria were not available (17). The European Committee on Antimicrobial Susceptibility Testing zone diameter interpretive standards were used for fusidic acid (18). The breakpoint used for interpretation of resistance to oxacillin was a zone of inhibition of ≥18 mm for *S. pseudintermedius* (19). Multidrug resistance (MDR) was defined as described by Magiorakos et al. (6), including isolates non-susceptible to at least 1 agent in ≥3 antimicrobial categories listed for *S. aureus*.

### Polymerase Chain Reaction (PCR) Testing for Virulence and Antimicrobial Resistance Genes

To extract DNA, a loopful of fresh staphylococcal colonies was homogenized in 90 μl sterile distilled water and 10 μl lysostaphin (1 mg/ml; Sigma-Aldrich Company Ltd., Gillingham, UK) and vortexed for 5 s. The suspension was incubated at 35°C for 10 min and then heated at 100°C for 10 min before adding 400 μl of sterile distilled water. DNA extractions were stored at 4 °C until used. Antimicrobial resistance genes investigated were *mec*A for methicillin resistance (20); *fusB, fusC*, and *fusD* for acquired low-level fusidic acid resistance (9); *ileS-2* for high-level mupirocin resistance (21, 22) and *qacA/B* and *smr* for efflux-mediated resistance to biocides (23). Virulence factors analyzed included leukocidin (encoded by *lukS*, or *lukF* genes) (24), exfoliative toxin (encoded by *siet, expA and expB* gene*s*) (25–27), and enterotoxin (encoded by *sec*_canine_ gene) (28). Biofilm forming capacity was investigated by screening for the presence of *bap, icaA*, or *icaD* genes (29, 30). Positive and negative controls were included in each polymerase chain reaction (PCR) assay. The primers, annealing temperature and expected DNA product size for each investigated gene are given in Table 1.

**Table 1:** Primers and annealing temperatures used to detect the carriage of virulence and resistance genes in the current study.

**Gene**	**Primers**	**Annealing temperature**	**Product size**	**References**
*lukS*	*fw*: 5′-TGTAAGCAGCAGAAAATGGGG-3′*rev*: 5′-GCCCGATAGGACTTCTTACAA-3′	57°C	503 bp	(24)
*lukF*	*fw*: 5′-CCTGTCTATGCCGCTAATCAA-3′*rev*: 5′-AGGTCATGGAAGCTATCTCGA-3′	57°C	572 bp	
*siet*	*fw*:5′-ATGGAAAATTTAGCGGCATCTGG-3′*rev*:5′-CCATTACTTTTCGCTTGTTGTGC-3′	56°C	359 bp	(25)
*sec_*canine*_*	*fw*:5′-GGGAAGCTTGTAATTTTGATATTCGCACT-3′*rev*:5′- CCCGGATCCTATCAAAATCGGATTAACA-3′	40°C	800 bp	(28)
*expA*	*fw*: 5′-GCGCGTCCTTCTGATCCAGAACT-3′*rev*: 5′-AACGTCCCCCTTTACCTACGTGAAT-3′	58°C	574 bp	(26)
*expB*	*fw*: 5′-GGGCATGCACATATGATGAAGCC-3′*rev*: 5′-CCAGATCTATCTTCTGATTCAGC-3′	50°C	843 bp	(27)
*fusB*	*fw*: 5′-CCGTCAAAGTTATTCAATCG-3′*rev*: 5′-ACAATGAATGCTATCTCGACA-3′	50°C	492 bp	(9)
*fusC*	*fw*: 5′-GGACTTTATTACATCGATTGAC-3′*rev*: 5′-CTGTCATAACAAATGTAATCTCC-3′	50°C	411 bp	
*fusD*	*fw*: 5′-AATTCGGTCAACGATCCC-3′*rev*: 5′-GCCATCATTGCCAGTACG-3′	57°C	465 bp	
*ileS*	*fw*: 5′-TATATTATGCGATGGAAGGTTGG-3′*rev*: 5′-AATAAAATCAGCTGGAAAGTGTTG-3′	57°C	458 bp	(21)
*smr*	*fw:* 5′-ATAAGTACTGAAGTTATTGGAAGT-3′*rev*: 5′-TTCCGAAAATGTTTAACGAAACTA-3′	48°C	285 bp	(23)
*qac A/B*	*fw*: 5′-GCTGCATTTATGACAATGTTTG-3′*rev*: 5′-AATCCCACCTACTAAAGCAG-3′	40°C	628 bp	
*icaA*	*fw:* 5′-CCTAACTAACGA AAGGTA G-3′ rev: 5′-AAG ATATAGCGA TAA GTG C-3′	49°C	1315 bp	(30)
*icaD*	*fw:* 5′-AAA CGT AAG AGA GGT GG-3′ rev: 5′-GGC AAT ATG ATC AAG ATA C-3′	49°C	381 bp	
*bap*	*fw:* 5′- CCCTATATCGAAGGTGTAGAATTG-3′*rev*: 5′-GCTGTTGAAGTTAATACTGTACCTGC-3′	62°C	971 bp	(29)

*fw, forward primer; rev, reverse primer; bp, base pair*.

### Statistical Analysis

Cross-tabulations were used to compare the frequency and prevalence of antimicrobial resistant isolates between the UK and Romania isolates for each of the studied phenotypes (MRSP, MSSP, and CoNS). Differences between countries were statistically analyzed using the Chi Square test of independence. When one or more cells in contingency tables contain <5 expected observations, the Fisher's exact test was used instead. Similarly, cross-tabulation, Chi-Square test/Fisher's exact test were used to compare the prevalence of each of the virulence and resistance genes investigated between countries. Statistical analysis was not possible if for example all isolates from both countries were PCR positive for a resistance or a virulence gene. Some isolates did not undergo antimicrobial susceptibility testing for some antimicrobials, and this was considered missing data. Data analyses were performed using R software version 3.5.1. (31). A critical probability of 0.05 was used for all analyses.

## Results

### Bacterial Isolates

*Staphylococcus* spp. isolates analyzed in the current study (*n* = 166) consisted of 115 CoPS identified as *S. pseudintermedius* based on the detection of species-specific *nuc* gene. These included 49 MSSP isolates from Romania (MSSP-RO), 40 MSSP isolates from the UK (MSSP-UK), 7 MRSP isolates from Romania (MRSP-RO) and 19 MRSP isolates from the UK (MRSP-UK). In addition, fifty-one CoNS isolates were identified which included 22 isolates from the UK (CoNS-UK) and 29 isolates from Romania (CoNS-RO). The CoNS-UK isolates included *S. epidermidis* (*n* = 8), *S. haemolyticus* (*n* = 8), *S. saprophyticus* (*n* = 2), *S. sciuri* (*n* = 2), and *S. warneri* (*n* = 2).

### Antimicrobial Susceptibility Testing

**Figure 1:**
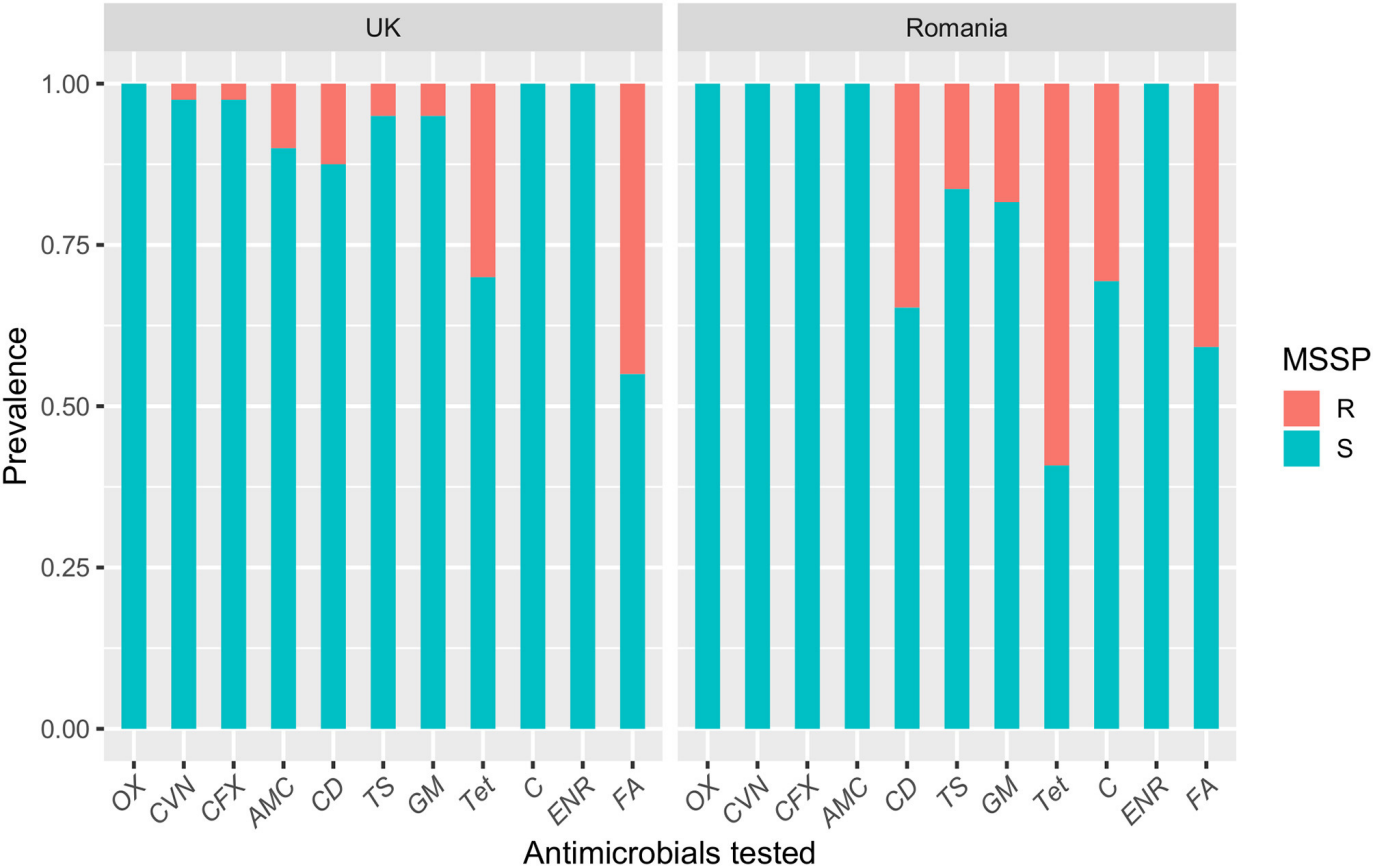
A bar plot showing the susceptibility profiles of methicillin-susceptible *Staphylococcus pseudintermedius* (MSSP) isolates from dogs with superficial pyoderma in the UK and Romania. OX, oxacillin; CVN, cefovecin; CFX, cefalexin; AMC, amoxicillin-clavulanic acid; CD, clindamycin; TS, trimethoprim-sulphamethoxazole; GM, gentamicin; Tet, tetracycline; C, chloramphenicol; ENR, enrofloxacin; FA, fusidic acid.

Table 2 compares the antimicrobial resistance profiles of MSSP, MRSP, and CoNS isolates from the UK and Romania. The Romanian MSSP isolates were more resistant toward tested antimicrobials compared with MSSP-UK isolates. This was statistically significant for clindamycin (34.7% vs. 12.5%; *P* = 0.03), tetracycline (59% vs. 30%; *P* = 0.01) and chloramphenicol (30.6% vs. 0%; *P* = 0.003). The MSSP-UK isolates showed greater resistance to amoxicillin-clavulanic acid (*P* = 0.08) compared with MSSP-RO isolates. In general, the 49 Romanian MSSP isolates analyzed showed total susceptibility to β-lactam antimicrobials and broader resistance profile to non β-lactam agents compared to the MSSP-UK isolates (Figure 1). Fusidic acid resistance, however, was commonly identified in both MSSP-UK (40.8%) and MSSP-RO (45%) isolates (*P* = 0.86).

**Table 2:** Antimicrobial resistance profiles of the Romanian and UK canine *Staphylococcus* spp. isolates.

**Antibiotic**	**MSSP**		***P***	**MRSP**		***P***	**CoNS**		***P***
	**Romania 49 (55.1)**	**UK 40 (54.9)**		**Romania 7 (27)**	**UK 19 (73)**		**Romania 29 (56.9)**	**UK 22 (43.1)**	
**β-lactams**									
Oxacillin	0 (0)	0 (0)	–	6 (85.7)	16 (84.2)	0.99	13 (44.8)	14 (63.6)	0.29
Cefovecin	0 (0)	1 (2.5)	–	1 (14.3)	9 (47.4)	0.19	6 (20.7)	9 (40.9)	0.21
Cefalexin	0 (0)	1 (2.5)	–	2 (28.6)	11 (57.9)	0.38	5 (17.2)	10 (45.5)	0.06
Amoxicillin-clavulanic acid	0 (0)	2 (10)	0.08	1 (14.3)	9 (47.4)	0.2	2 (6.9)	9 (90)	<0.001
**Non** **β-lactams**									
Clindamycin	17 (34.7)	5 (12.5)	0.03	7 (100)	13 (68.4)	0.15	7 (24.1)	8 (36.4)	0.52
Trimethoprim-sulphamethoxazole	8 (16.3)	2 (5)	0.18	5 (71.4)	13 (68.4)	0.99	1 (3.5)	6 (27.3)	0.03
Gentamicin	9 (18.4)	2 (5)	0.10	5 (71.4)	8 (42.1)	0.38	1 (3.5)	8 (36.4)	0.03
Tetracycline	29 (59.2)	12 (30)	0.01	7 (100)	8 (72.7)	0.24	17 (58.6)	9 (40.9)	0.33
Chloramphenicol	15 (30.6)	0 (0)	0.003	1 (14.3)	6 (31.6)	0.62	3 (10.3)	0 (0)	0.55
Enrofloxacin	0 (0)	0 (0)	–	2 (28.6)	10 (52.6)	0.4	5 (17.2)	4 (40)	0.2
Fusidic acid	20 (40.8)	18 (45)	0.86	1 (14.3)	11 (100)	<0.001	12 (41.4)	14 (63.6)	0.19
MDR	16 (32.7)	3 (7.5)	0.004	7 (100)	18 (94.7)	0.99	10 (34.5)	12 (54.6)	0.25

*The data is presented as number (percentage) of isolates exhibiting resistance to each tested antimicrobial. P-values are from either Fisher's exact test or Chi-Square test for count data*.

*MSSP, methicillin susceptible Staphylococcus pseudintermedius; MRSP, methicillin-resistant Staphylococcus pseudintermedius; CoNS, coagulase-negative staphylococci; MDR, multidrug resistant*.

**Figure 2:**
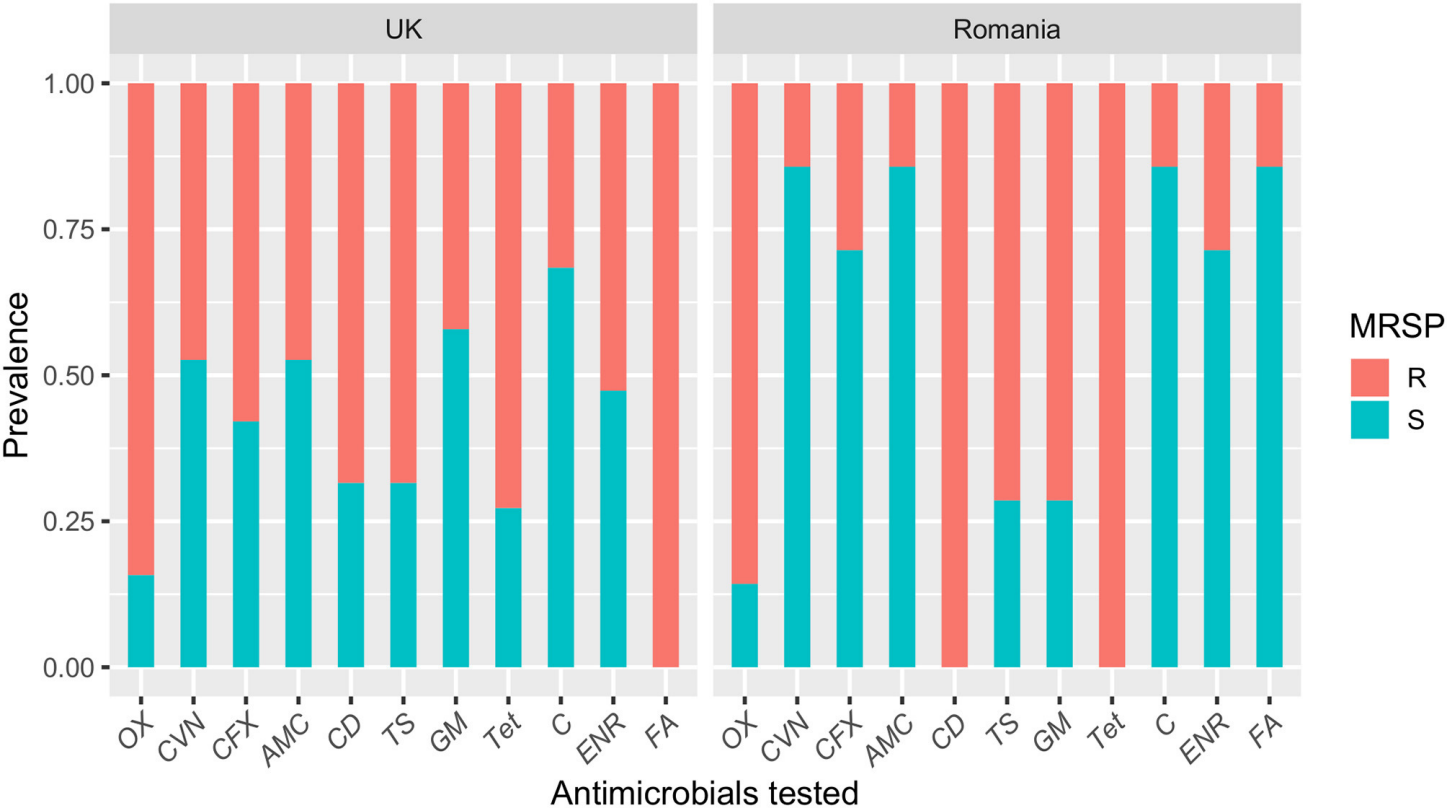
A bar plot showing the susceptibility profiles of methicillin-resistant *Staphylococcus pseudintermedius* (MRSP) isolates from dogs with superficial pyoderma in the UK and Romania. OX, oxacillin; CVN, cefovecin; CFX, cefalexin; AMC, amoxicillin-clavulanic acid; CD, clindamycin; TS, trimethoprim-sulphamethoxazole; GM, gentamicin; Tet, tetracycline; C, chloramphenicol; ENR, enrofloxacin; FA, fusidic acid.

**Figure 3:**
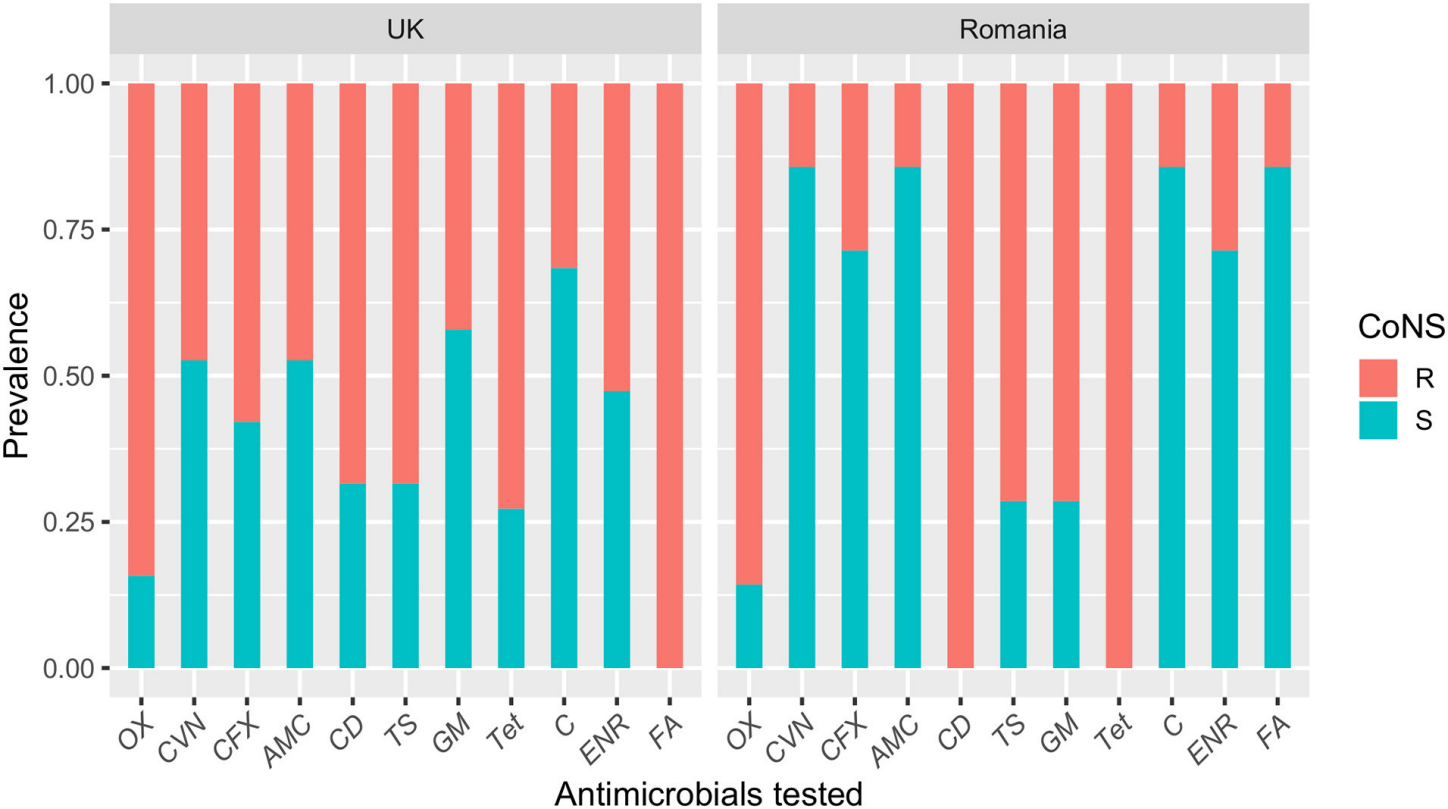
A bar plot showing susceptibility profiles of coagulase-negative staphylococci (CoNS) isolates from dogs with superficial pyoderma in the UK and Romania. OX, oxacillin; CVN, cefovecin; CFX, cefalexin; AMC, amoxicillin-clavulanic acid; CD, clindamycin; TS, trimethoprim-sulphamethoxazole; GM, gentamicin; Tet, tetracycline; C, chloramphenicol; ENR, enrofloxacin; FA, fusidic acid.

Resistance to the antimicrobials tested was common amongst MRSP-UK and MRSP-RO isolates and most isolates were multi-drug resistant (100% and 94.7%, respectively) (Figure 2). Fusidic acid resistance was significantly higher in MRSP-UK compared to MRSP-RO isolates (100% vs. 14.2%; *P* < 0.001). The prevalence of antimicrobial resistance in CoNS isolates investigated was greater in those originating from the UK dogs compared with Romanian isolates for most tested antimicrobials (Figure 3). These differences were statistically significant for amoxicillin-clavulanic acid (90% vs. 6.2%; *P* < 0.001), trimethoprim-sulphamethoxazole (27.3% vs. 3.4%; *P* = 0.03) and gentamicin (36.4% vs. 3.4%; *P* = 0.03). The prevalence of MDR CoNS isolates was higher in the UK isolates although this difference was not statistically significant (54.6% vs. 34.5% for the CoNS-UK and CoNS-Ro, respectively; *P* = 0.25).

### Presence of Resistance and Virulence Genes

All MRSP-RO and MRSP-UK isolates were positive for the leukocidin genes *lukS* and *lukF*, and the exfoliative gene *siet*, whilst 98 and 97.5% of MSSP-RO and MSSP-UK isolates carried these genes, respectively (Table 3). The *sec*_*canine*_ enterotoxin gene was only identified in MSSP isolates (12.2 and 10% of MSSP-RO and MSSP-UK isolates, respectively). Overall, 16 (9.64%) *Staphylococcal* isolates carried the *expA* exfoliative gene (8 MSSP-RO, 5 MSSP-UK and 3 CoNS-Ro isolates) whereas the *expB* exfoliative gene was carried by 6.25% (*n* = 9) of isolates (5 MSSP-RO and 4 MRSP-RO isolates). None of the MRSP-UK or CoNS-UK isolates carried the exfoliative genes *expA* and *expB* or the *sec*_*canine*_ enterotoxin gene. Greater numbers of CoNS-RO isolates carried the *lukS* (*P* = 0.06), *lukF* (*P* = 0.06), *siet* (*P* = 0.01), *and expA* (*P* = 0.25) virulence genes compared with CoNS-UK isolates. Biofilm producing genes were rare both among the *S. pseudintermedius* (1 MSSP-RO with *icaD* and 1 MRSP-UK with *bap*) and the CoNS isolates (1 CoNS-RO with *icaA*, 2 with *icaD*, and 3 CoNS-UK with *icaA*).

**Table 3:** Antimicrobial/antiseptic resistance and virulence gene profiles of the Romanian and UK *Staphylococcus* spp. canine isolates. The data is presented as number (percentage) of isolates exhibiting virulence genes.

**Gene**	**MSSP**		***P***	**MRSP**		***P***	**CoNS**		***P***
	**Romani 49 (55.1)**	**UK 40 (44.9)**		**Romania 7 (27)**	**UK 19 (73)**		**Romania 29 (56.9)**	**UK 22 (43.1)**	
*luk*S	48 (98)	39 (97.5)	–	7 (100)	19 (100)	–	5 (17.2)	0 (0)	0.06
*luk*F	48 (98)	39 (97.5)	–	7 (100)	19 (100)	–	5 (17.2)	0 (0)	0.06
*siet*	49 (100)	39 (97.5)	–	7 (100)	19 (100)	–	7 (25)	0 (0)	0.01
*exp*A	8 (16.3)	5 (12.5)	0.84	0 (0)	0 (0)	–	3 (10.3)	0 (0)	0.25
*expB*	5 (10.2)	0 (0)	0.06	4 (57.1)	0 (0)	0.002	0 (0)	0 (0)	–
*sec*_canine_	6 (12.2)	4 (10)	0.99	0 (0)	0 (0)	–	0 (0)	0 (0)	–
*fus*B	0 (0)	0 (0)	–	0 (0)	0 (0)	–	0 (0)	12 (54.5)	<0.001
*fus*C	0 (0)	0 (0)	–	0 (0)	3 (15.8)	0.54	2 (6.9)	0 (0)	0.5
*fus*D	0 (0)	0 (0)	–	0 (0)	0 (0)	–	1 (3.5)	2 (9.1)	0.56
*ileS*	0 (0)	0 (0)	–	0 (0)	0 (0)	–	1 (3.4)	5 (22.7)	0.07
*mec*A	0 (0)	0 (0)	–	7 (100)	19 (100)	–	7 (24.1)	13 (59.1)	0.02
*smr*	0 (0)	0 (0)	–	0 (0)	1 (5.3)	–	1 (3.5)	2 (9.1)	0.57
*qacA/B*	1 (2.0)	0 (0)	–	0 (0)	1 (5.3)	–	3 (10.3)	13 (59.1)	<0.001
*icaA*	0 (0)	0 (0)	–	0 (0)	0 (0)	–	1 (3.5)	3 (13.6)	0.3
*icaD*	1 (2.0)	0 (0)	–	0 (0)	0 (0)	–	2 (6.9)	0 (0)	0.5
*bap*	0 (0)	0 (0)	–	0 (0)	1 (5.3)	–	0 (0)	0 (0)	–

*P-values are from either Fisher's exact test or Chi-Square test for count data*.

*MSSP, methicillin susceptible Staphylococcus pseudintermedius; MRSP, methicillin-resistant Staphylococcus pseudintermedius; CoNS, coagulase-negative staphylococci*.

Fusidic acid resistance gene prevalence was 15.8% (*n* = 3/19) in MRSP-UK, 63.63% (*n* = 14/22) in CoNS-UK and 10.3% (*n* = 3/29) in CoNS-RO isolates. None of the MSSP isolates either from the UK or Romania, carried the fusidic acid resistance genes. A total of 48.1 % (*n* = 76) of all tested isolates were phenotypically resistant to fusidic acid, of which 12.7% (*n* = 20) carried fusidic acid resistance genes (*fus*B, *fus*C, or *fus*D). Mupirocin resistance gene (*ileS-2)* was detected in five CoNS-UK isolates (23%, 5/22) and one CoNS-RO isolate (3.4%, 1/29). Significantly greater numbers of CoNS-UK isolates were identified with *fus*B (*P* < 0.001), *mec*A (*P* = 0.02) and *qacA/B* (*P* < 0.001) antimicrobial resistance genes. The *mec*A gene was carried by 46 isolates: 19 MRSP-UK (100%), 7 MRSP-RO (100%), 7 methicillin-resistant CoNS-RO (24.1%), and 13 methicillin-resistant CoNS-UK (59.1%) isolates. Plasmid-mediated antiseptic resistance (*qacA/B* and *smr*) genes were detected in 1 MSSP-RO isolate (carried the *qacA/B gene*), 1 MRSP-UK (carried both genes), 4 CoNS-RO isolates (1 isolate carried *smr* and 3 had *qacA/B* gen*e*), and 15 CoNS-UK isolates (2 carried *smr* gene and 13 had *qacA/B* gene).

## Discussion

The aim of the current study was to compare the antimicrobial susceptibility profiles and virulence potential of mucosal and cutaneous MSSP, MRSP, and CoNS from dogs diagnosed with superficial pyoderma originating from two European geographic regions: eastern Romania and the UK. Notably, in this study *S. aureus* was not identified amongst resident flora of the investigated patients, which is consistent with numerous reports recognizing *S. pseudintermedius* as the leading cause of canine pyoderma (32, 33). Overall, differences in the antimicrobial resistance patterns have been identified, with the most important within the MSSP group. The Romanian MSSP isolates were more frequently identified with phenotypic resistance to gentamicin, clindamycin, chloramphenicol, tetracycline, trimethoprim-sulphamethoxazole and surprisingly, 100% ß-lactam susceptibility. In contrast, MSSP-UK isolates showed variable levels of resistance to ß-lactam antibiotics (2.5–10%), possibly reflecting a more consistent use of cephalexin and amoxicillin-clavulanic acid as a first-line treatment for skin disease in dogs in the UK. However, other factors such as the genetic background of the isolates can also contribute to these differences. Previous studies have shown that successful lineages of *S. pseudintermedius* with specific antimicrobial resistance traits can emerge in different geographical regions (34, 35) and this could be the case in the current study. However, MLST analysis or other molecular typing was not performed which is the main limitation of this study, as this would have allowed for better comparison of the genetic background of the isolates collected from these two different European geographical regions.

As expected, of the six *Staphylococcus* spp. groups examined in the current study, overall resistance was greatest amongst the methicillin-resistant group, in both MRSP-UK and MRSP-RO isolates. Interestingly, only 85.7 and 84.2% of the MRSP-RO and MRSP-UK isolates exhibited resistance to oxacillin, the preferred agent for detecting methicillin resistance in *S. pseudintermedius* (17), despite the 100% carriage of *mecA* gene in both groups. Black et al. (36) reported significant differences in *mecA* expression of MRSP clones from different geographic regions. These authors showed that isolates of multi-locus sequence type (MLST) 68 and 71, which predominate in North American and Europe, respectively, have dissimilar phenotypes when exposed to oxacillin *in vitro*, exhibiting either a slow or robust response with regard to oxacillin-induced *mecA* expression (36).

Similarly, a number of the CoNS isolates (1 CoNS-RO and 6 CoNS-UK) analyzed were phenotypically oxacillin resistant despite the fact that they did not carry the *mec*A gene, likely owing their oxacillin-resistance to β-lactamase hyper-production. This finding is consistent with a previous study investigating antimicrobial resistance patterns of staphylococcal isolates from healthy dogs in the USA (37). Moreover, phenotypic oxacillin resistance amongst *mecA*-negative CoNS could also be due to the presence of an alternative methicillin resistance gene such as *mecC* (38), which was not investigated in our isolates.

The majority of *S. pseudintermedius* isolates investigated in the current study carried the leukocidin genes *luk*S and *luk*F and the exfoliative gene *siet*. Similarly, high prevalence (up to 100%) of these virulence genes amongst *S. pseudintermedius* isolates with no apparent difference between MSSP and MRSP has been reported (5, 39, 40). A small proportion of MSSP isolates carried *exp*A, *expB*, and *sec*_canine_ genes in the current study; this is consistent with other studies where a variable prevalence of these genes was reported [12.6% (41) and of 24.3% (42) of *sec*_canine_ gene]. The prevalence of *expB* and *expA* genes reported in the current study was lower than the *expB* prevalence of 23.2% reported in clinical *S. pseudintermedius* isolates from dogs with superficial pyoderma (27) and the *expA* gene prevalence of 31% reported in canine *S. pseudintermedius* isolates from Spain (5). The *expA* (formerly known as *exi*) and *expB* genes encode exfoliating toxins (43) that were shown to be associated with subcorneal clefts, erythema, vesicles, and erosions when purified and injected into canine skin (27, 44).

The current study reported high prevalence of fusidic acid resistance encoded by *fus*B genes (54.5%) amongst CoNS-UK isolates and this correlated with the presence of phenotypic resistance except for two isolates. However, overall phenotypic resistance to fusidic acid (48.1%) did not correlate with carriage of genes encoding for fusidic acid resistance (12.7%) in this study. Other mechanisms of resistance, such as chromosomal mutations (*fusA*) have been shown to be also involved (9). More recently, Frosini et al. (45) have shown that increased minimum inhibitory concentrations (MICs) to fusidic acid are frequently associated with carriage of *fus*C as well as *fus*A chromosomal mutations. However, fusidic acid is only authorized for topical use in the treatment of canine pyoderma in the UK (46) and may therefore achieve high concentrations at the site of infection overcoming resistance.

Screening for genes known to encode efflux-mediated resistance to biocides identified one MSSP-RO *qacA/B* positive and one MRSP-UK *qacA/B* and *smr* positive isolate. These results are consistent with those from previous studies which reported either no *S. pseudintermedius* isolates positive for the *qacA/B* or the *smr* genes (11) or only identified a single MSSP isolate carrying the *qacA/B* gene (10). However, more recently, two equine MRSA isolates from Australia were found to harbor *qacA/B* genes and the study also showed that some MRSA lineages (i.e., ST71) are more likely to carry *qac* genes (47). Although in our study carriage of *qacA/B* was significantly higher in the CoNS-UK isolates compared to the CoNS-RO isolates, Frosini et al. (45) has shown that carriage of the *qacA/B* gene tends not to correlate with a high chlorhexidine MIC, which brings into question the clinical significance of *qacA/B* carriage. Nevertheless, the emergence of biocide resistant *S. pseudintermedius* strains, particularly if concurrently methicillin resistant, would severely limit therapeutic options and potentiate clinical outbreaks as already demonstrated for mupirocin resistance (48) and chlorhexidine resistance (49) amongst *S. aureus* in humans.

The broad antimicrobial resistance to non β-lactam antimicrobials identified amongst the MSSP-RO isolates is surprising and could reflect differences in the antimicrobial prescribing and usage patterns between these countries. Although the treatment history of the dogs included in the current study was unknown, the cases were investigated at a referral hospital and it is likely that they may have received antimicrobials in first opinion practices. Similar to the situation in human medicine in Romania where self-medication is prevalent (50), antimicrobial usage in companion animals is not as strictly monitored as in other European countries and this is demonstrated by the fact that until recently antimicrobials could be purchased over the counter from veterinary pharmacies in Romania, which can contribute to the problem of AMR.

To our knowledge, this is the first study to compare *S. pseudintermedius* isolates from dogs with superficial pyoderma originating from two different geographic regions in Europe, where treatment protocols are likely to be different due to compliance in relation to implementation of antimicrobial stewardship guidelines, availability, authorization, and cost. Our findings suggest that further studies on antimicrobial use and prescribing patterns, as well as rigorous surveillance of AMR in companion animals in Romania is critical for reducing the overall burden of resistance genes circulating and which can be exchanged between humans and animals. In addition, there is a recent trend for importation of companion animals from Eastern European countries (including Romania) and although the risk of zoonotic disease transmission has been highlighted (51), the risk for antimicrobial resistant bacteria spread through companion animal importation is also concerning and requires increased awareness.

## Data Availability Statement

All datasets generated for this study are included in the article/Supplementary Material.

## Ethics Statement

The animal study was reviewed and approved by University of Liverpool Ethics Committee, the Romanian Faculty of Veterinary Science Ethics and Deontology Committee. Written informed consent was obtained from the owners for the participation of their animals in this study.

## Author Contributions

OH contributed to the laboratory experiments, including susceptibility testing and molecular analysis, and drafted the manuscript. DT contributed to study design, supervised laboratory work at University of Liverpool (UoL), and read and revised the manuscript. SS contributed to the laboratory work including susceptibility testing and molecular analysis, performed the statistical analyses, and read and revised the manuscript. IM contributed to PCR experiments, molecular analysis, and read the manuscript. RM and IL carried out laboratory work including phenotypic and biochemical characterization of *S. pseudintermedius* isolates at the Faculty of Veterinary Medicine Iasi (FMV Iasi) and read the manuscript. MM coordinated laboratory work at the FMV Iasi and read and revised the manuscript. GS coordinated patient and isolates selection at the FMV Iasi and read and revised the manuscript. VS contributed to the study design, supervised laboratory work at UoL, coordinated patient selection at UoL, and read and revised the manuscript. All authors contributed to the article and approved the submitted version.

## Conflict of Interest

The authors declare that the research was conducted in the absence of any commercial or financial relationships that could be construed as a potential conflict of interest.
